# The Smc complexes in DNA damage response

**DOI:** 10.1186/2045-3701-2-5

**Published:** 2012-02-27

**Authors:** Nan Wu, Hongtao Yu

**Affiliations:** 1 Department of Pharmacology, Howard Hughes Medical Institute, 6001 Forest Park Road, Dallas, TX 75390, USA

**Keywords:** Cohesin, Condensin, Smc5, Smc6, homologous recombination, DNA repair, DNA damage checkpoint, rDNA, SUMO

## Abstract

The structural maintenance of chromosomes (Smc) proteins regulate nearly all aspects of chromosome biology and are critical for genomic stability. In eukaryotes, six Smc proteins form three heterodimers--Smc1/3, Smc2/4, and Smc5/6--which together with non-Smc proteins form cohesin, condensin, and the Smc5/6 complex, respectively. Cohesin is required for proper chromosome segregation. It establishes and maintains sister-chromatid cohesion until all sister chromatids achieve bipolar attachment to the mitotic spindle. Condensin mediates chromosome condensation during mitosis. The Smc5/6 complex has multiple roles in DNA repair. In addition to their major functions in chromosome cohesion and condensation, cohesin and condensin also participate in the cellular DNA damage response. Here we review recent progress on the functions of all three Smc complexes in DNA repair and their cell cycle regulation by posttranslational modifications, such as acetylation, phosphorylation, and sumoylation. An in-depth understanding of the mechanisms by which these complexes promote DNA repair and genomic stability may help us to uncover the molecular basis of genomic instability in human cancers and devise ways that exploit this instability to treat cancers.

## Introduction

**Figure 1:**
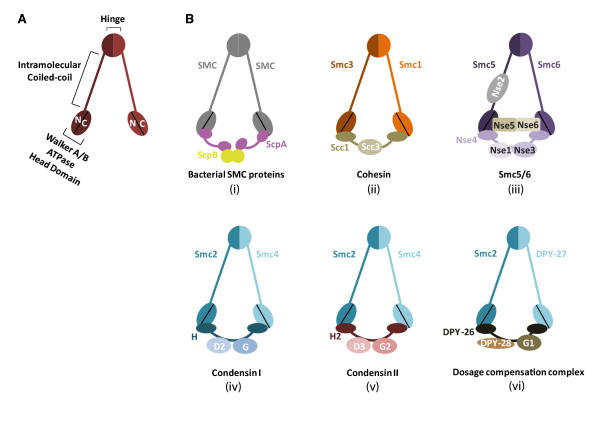
**Architecture of the Smc complexes**. (**A**) The core of each Smc complex is formed by two Smc proteins. Each Smc protein contains an ATPase head domain, a hinge domain, and an intramolecular antiparallel coiled coil that connects the two. The hinge domain mediates the dimerization of Smc proteins. (**B**) Various Smc complexes found in bacteria and eukaryotes. Each Smc complex is composed of a specific Smc dimer and several non-Smc subunits. (i) The bacterial Smc complex from *Bacillus subtilis*. ScpA connects the two ATPase heads of the Smc homodimer. (ii) The Smc1/3 cohesin complex. (iii) The Smc5/6 complex. (iv) The condensin I complex. H, D2, and G stand for CAP-H, CAP-D2, and CAP-G, respectively. (v) The condensin II complex. H2, D3, and G2 stand for CAP-H2, CAP-D3, and CAP-G2, respectively. (vi) The condensin-like dosage compensation complex in *C. elegans*. DPY-27 is an Smc4 variant.

The highly conserved structural maintenance of chromosomes (Smc) proteins regulate chromosome architecture and organization from bacteria to human. Most prokaryotes have a single Smc protein which forms a homodimer, while there are at least six Smc family members, Smc1-6, that form three heterodimers in eukaryotic organisms [1] (Figure 1). Smc1 and Smc3 form the core of the cohesin complex which maintains sister-chromatid cohesion during mitosis to ensure accurate chromosome segregation [2]. Smc2 and Smc4 constitute the condensin complexes that promote chromosome condensation [3]. Smc5 and Smc6 form a complex that plays critical roles in DNA repair [4,5].

The Smc proteins contain about 1,000 amino acids and share similar domain structures. The ATPase domain of each Smc protein is separated into N- and C-terminal halves by a long linker. The two nucleotide-binding Walker A and Walker B motifs reside in the two different ATPase halves. The Smc linker folds into an intramolecular antiparallel coiled coil and allows the N-terminal ATPase half of an Smc protein to fold back to its C-terminal ATPase half and create a single globular ATPase head (Figure 1). The hinge domain at one end of the coiled coil mediates the heterodimerization of eukaryotic Smc proteins [1,6,7]. The two ATPase heads at the other end of the coiled coil can transiently interact with each other to bind and hydrolyze ATP. As revealed by electron microscopy, the Smc heterodimers can adopt different conformations, including V-shaped dimer and ring-like structures, possibly depending on the nucleotide-binding states of their ATPase heads [8,9]. Each Smc heterodimer associates with non-Smc subunits to form functional Smc complexes.

The genomic DNA with a cell experiences many types of damage daily. These damages can result from exogenous factors, such as ultraviolet (UV) radiation, ionizing radiation (IR), and chemical carcinogens, or from endogenous factors, such as stalled replication forks due to replication stress. In response to DNA damage, cells elicit elaborate DNA damage responses. For example, DNA damage checkpoints arrest cell cycle progression at various points, thus affording more time for cells to execute DNA repair. Failure to properly repair DNA damage can result in cell death or genomic instability which may eventually lead to cancer [10].

The role of the Smc5/6 complex in DNA repair has long been appreciated. Emerging evidence in recent years has established that, in addition to their fundamental roles in chromosome segregation and organization, cohesin and condensin are also required for DNA damage checkpoints and DNA repair. In this review, we summarize the roles of these three Smc complexes in DNA damage response and the maintenance of genomic stability. We note that Rad50 is structurally related to the Smc proteins and has well-established roles in DNA repair as a subunit of the Mre11-Rad50-Nbs1 (MRN) complex. Because the MRN complex has been the subject of recent reviews [11], we will not focus on it in the current review.

## 1. The Smc1/3 cohesin complex

The cohesin complex is composed of four evolutionarily conserved subunits, Smc1, Smc3, and two non-Smc proteins named Scc1 and Scc3 [1,12] (Table 1). Vertebrate cells contain two Scc3 proteins, called SA1 and SA2. The N- and C-terminal regions of Scc1 link the head domains of Smc3 and Smc1, respectively, forming a tripartite ring. Scc3 is predicted to be a HEAT repeat-containing protein and interacts with Scc1 to further strengthen the ring structure of cohesin. Cohesin has been proposed to topologically embrace DNA and chromatids inside its ring. Other cohesin-binding proteins include Wapl, Pds5, and sororin (in metazoans) that associate with cohesin in a sub-stoichiometric manner. Their interactions with cohesin are regulated during the cell cycle. Wapl negatively regulates cohesin association with chromatin while sororin stabilizes cohesin on chromatin. Pds5 appears to have dual functions in cohesin regulation.

**Table 1:** Components of the Smc complexes and regulatory proteins in different organisms

	*S. cerevisiae *	*S. pombe *	*H. sapiens *
**Cohesin**	Smc1	Psm1	Smc1
	
	Smc3	Psm3	Smc3
	
	Mcd1/Scc1	Rad21	Scc1/Rad21
	
	IRR1/Scc3	Psc3	SA1/STAG1,SA2/STAG2

**Cohesin regulators**	Scc2	Mis4	NIPBL
	
	Scc4	Ssl3	MAU2/Scc4
	
	Eco1/Ctf7	Eso1	EFO1/ESCO1,EFO2/ESCO2
	
	Pds5	Pds5	Pds5A, Pds5B
	
	Rad61	Wpl1	Wapl
	
	-	-	Sororin

**Condensins**	Smc2 (I&II)	Cut14	CAP-E
	
	Smc4 (I&II)	Cut3	CAP-C
	
	Brn1	Cnd2	CAP-H (I)
	
	Ycs4	Cnd1	CAP-D2 (I)
	
	Ycs5	Cnd3	CAP-G (I)
	
	-	-	CAP-D3 (II)
	
	-	-	CAP-G2 (II)
	
	-	-	CAP-H2 (II)

**The Smc5/6 complex**	Smc5	Spr18/Smc5	Smc5
	
	Rhc18/Smc6	Rad18/Smc6	Smc6
	
	Nse1	Nse1	Nse1
	
	Mms21/Nse2	Nse2	Nse2
	
	YDR228W/Nse3	Nse3	Nse3
	
	Qri2/Nse4	Rad62/Nse4	Nse4
	
	YML023C/Nse5	Nse5	-
	
	Kre29/Nse6	Nse6	-

### 1.1. Cohesin and sister-chromatid cohesion

The major function of cohesin, as its name indicates, is to regulate sister-chromatid cohesion. Cohesin is loaded by the cohesin loader Scc2/4 complex in telophase and G1 prior to DNA replication. The loaded cohesin then becomes cohesive during DNA replication and has been proposed to topologically embrace both sister chromatids inside its ring to establish sister-chromatid cohesion. The mechanism by which cohesin is converted to the cohesive state during DNA replication is not completely understood, but it requires the acetylation of Smc3 by the Eco1 family of acetyltransferases [13-16]. In vertebrates, Smc3 acetylation enables the binding of sororin to Pds5, which counteracts Wapl's ability to remove cohesin from chromatin [17-19]. Because sororin homologs have not been found in yeast, how Smc3 acetylation makes cohesin refractory to Wapl in yeast remains to be determined.

Timely dissolution of sister-chromatid cohesion is required for proper chromosome segregation in mitosis. In yeast, cohesin is cleaved by the protease separase at the metaphase-anaphase transition to trigger sister-chromatid separation. In humans, most cohesin on chromatid arms is removed by Wapl in prophase, and this process is facilitated by Plk1-dependent phosphorylation of SA2 [20-22]. Only a small amount of cohesin remains associated with the centromeres and is protected from Wapl and Plk1 by the shugoshin-PP2A complex [23-25]. This centromeric pool of cohesin is cleaved by separase at metaphase to allow sister-chromatid separation [26].

### 1.2. Cohesin and DNA repair

**Figure 2:**
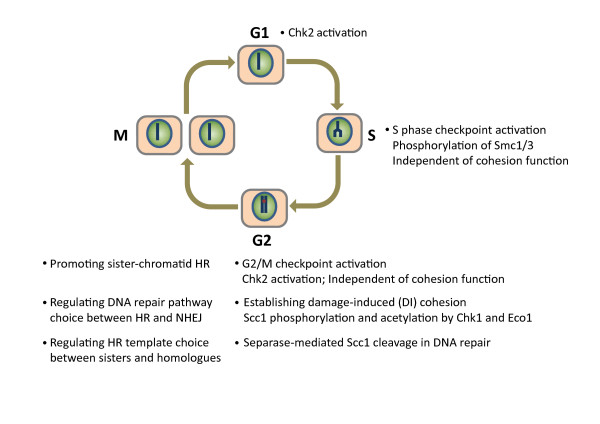
**Functions of cohesin in DNA damage response during the cell cycle**. The function of cohesin in promoting DSB repair through sister-chromatid homologous recombination (HR) has been established in multiple organisms ranging from yeast to man. Most of the other proposed functions are only demonstrated in specific organisms, and their evolutionary conservation needs to be further tested.

In addition to its function in sister-chromatid cohesion, cohesin plays critical roles in DNA damage response (Figure 2). In fact, cohesin's role in DNA repair was discovered before the discovery of its function in sister-chromatid cohesion. The cohesin subunit Scc1 was first identified as Rad21, whose mutation rendered *S. pombe *cells hypersensitive to UV or IR [27,28]. Later studies further confirmed a role of cohesin in DNA repair in several organisms, including *S. cerevisiae*, chicken, and humans [29-33]. These studies further pinpointed a specific function of cohesin in DNA double-strand break (DSB) repair through homologous recombination (HR).

In *S. cerevisiae*, cohesin and its positive regulators, including Scc2, Pds5, and Eco1, are all required for DSB repair during G2 [29]. Thus, it is not the cohesin complex per se but rather damage-induced functional sister-chromatid cohesion that is required for DNA repair. Moreover, in addition to observing the expected premature sister-chromatid separation phenotype in a conditional Scc1-deficient chicken DT40 cell line, Sonoda *et al. *also observed a marked defect in DNA repair in these cells [30]. The Scc1-deficient cell line exhibited increased chromosome aberrations in S/G2 and a reduced frequency of sister-chromatid exchange (SCE). Finally, human cells with cohesin subunits depleted by RNA interference (RNAi) also exhibited DNA repair and SCE defects [32-34]. Taken together, these data establish a crucial role for cohesin in DSB repair through sister-chromatid HR during S/G2 phases of the cell cycle.

What is the function of cohesin in HR? HR-mediated DSB repair requires an undamaged DNA template. During the mitotic cell cycle, sister chromatids are the preferred template for HR, as HR between homologues may lead to the loss of heterozygosity. It is generally believed that sister-chromatid cohesion at or near a DSB helps to keep the DSB and the undamaged sister chromatid at close proximity, thereby promoting strand invasion and sister-chromatid HR. Although this notion makes intuitive sense, it remains to be formally tested experimentally. Consistent with a specific requirement for cohesin in sister-chromatid HR, cohesin is not required for certain forms of HR in *S. cerevisiae*, including intrachromosomal gene conversion [35]. Cohesin is also not required for processes that occur in all forms HR, such as end resection and the formation of single-strand DNA at DSBs. Furthermore, cohesin suppresses DNA damage-induced recombination between homologous chromosomes in yeast [36]. Finally, cohesin coordinates DSB repair pathway choice between HR and non-homologous end joining (NHEJ) through interaction with the Rad52 pathway [31]. These results are consistent with a specific role of cohesin in sister-chromatid HR. It is possible that the elevation of NHEJ and HR between homologues seen in cohesin-deficient cells is simply due to repair pathway competition. Alternatively, cohesin may actively suppress NHEJ and HR between homologues through unknown mechanisms.

### 1.3. Recruitment of cohesin to DSBs

A direct role of cohesin in DNA repair is further supported by its recruitment to DSBs, which was shown by both immunofluorescence (IF) and chromatin immunoprecipitation (ChIP). First, the Yokomori group detected the recruitment of cohesin to laser-induced DNA damage sites in human cells [37]. A laser microbeam was used to create DNA damage at discrete sites in the cell, and the recruitment of DNA repair proteins to these sites was monitored by IF. Shortly after radiation, cohesin and the MRN component Mre11 were detected at these sites. The recruitment of cohesin to these DNA damage foci was limited to S/G2 phases of the cell cycle, and dependent on Mre11 and Rad50. Furthermore, Rad50 was co-immunoprecipitated with cohesin subunits from human cells in S/G2, supporting a model in which MRN recruits cohesin to DSBs.

The recruitment of cohesin to DSBs is further supported by ChIP studies in yeast and human cells [34,35,38]. In these cases, specific restriction endonucleases were used to cut defined sites in the genome to generate DSBs. In the study by the Koshland group [35], cohesin enrichment around a DSB was detected with ChIP in G2/M phases, but not in G1 phase, of the cell cycle in *S. cerevisiae*, and required Scc2/4. In addition, proteins with well-known functions in HR, such as Mec1, Tel1, Rad53, Mre11, and γH2A, are all required for the establishment of the DSB-specific cohesin domain. In an accompanying study, the Sjögren group reported that cohesin was recruited to 50-kb genomic regions flanking a DSB induced during G2/M in yeast [38]. They also showed that Scc2/4 was necessary for cohesin accumulation and DNA repair. Curious of the molecular function of cohesin recruitment to DSBs, the authors designed an elegant experiment to test whether functional cohesion was generated after induction of DSB. Indeed, they showed that cohesin recruited to the DSB was able to establish sister-chromatid cohesion in G2 phase after DNA replication. Finally, using ChIP, our lab showed that cohesin was also recruited to an I-SceI-induced DSB in human cells [34]. Thus, cohesin recruitment to DSBs is a conserved process of the DNA damage response in organisms from yeast to man.

### 1.4. DNA damage-induced sister-chromatid cohesion

Both the Sjögren and Koshland groups found in yeast that DNA damage induced sister-chromatid cohesion not only at the DSB site but also throughout the genome after genome duplication in G2 [39,40]. This damage-induced (DI) cohesion was controlled by the DNA damage response factors (Mec1, Tel1, Mre11, and γH2A) and cohesin regulators (Scc2, Eco1, and Smc6), but was independent of DNA replication. Overexpression of Eco1, but not an Eco1 mutant lacking its acetyltransferase activity, bypassed the requirement for DSBs in DI-cohesion generation in G2/M [40]. These results suggested that the activity of Eco1 was limiting in undamaged G2/M cells, and DNA damage response reactivated its activity.

How does DNA damage response augment Eco1's activity after DNA replication? What is the upstream signal? Does the signal regulate Eco1 or its substrate? What is the Eco1 substrate? Excellent studies by the Koshland group began to answer these questions [41,42]. They showed that phosphorylation of the cohesin subunit Scc1 (also known as Mcd1) by Chk1 at S83 was critical for DI cohesion. Substitution of S83 to alanine (S83A) inhibited DI cohesion, while substitution of S83 to aspartic acid (S83D) to mimic phosphorylation generated cohesion during G2/M, even in absence of a DSB or Chk1. Functional Eco1 was, however, still required for cohesion in S83D cells. Moreover, genetic evidence suggested that K84 and K210 of Scc1 were the Eco1-dependent acetylation sites in response to the DSB. Mutation of these two lysines to glutamine to mimic acetylation bypassed the requirement for the DSB or the acetyltransferase activity of Eco1 in DI cohesion. Furthermore, Smc3 acetylation by Eco1 was uniquely required for S-phase cohesion, but not for DI cohesion. Both Smc3 acetylation and Scc1 acetylation appeared to counteract the function of Wapl to establish cohesion. Collectively, their results support a model in which phosphorylation of Scc1 at S83 by Chk1 makes Scc1 a better substrate for Eco1, which then acetylates K84 and K210 of Scc1 to establish DI cohesion (Figure 2). Eco1 thus has distinct substrates in S-phase cohesion and DI cohesion. Future biochemical experiments are needed to confirm the phosphorylation and acetylation of Scc1 in response to the DSB.

So far, direct evidence for DI cohesion is only available in budding yeast. Several lines of indirect evidence suggest that DI cohesion might be a conserved mechanism in higher organisms. First, the critical cohesion-establishment factor, sororin, is required for efficient DSB repair during G2 in HeLa cells [19]. Sororin RNAi cells showed a marked increase of DNA breaks, compared to control cells. In addition, decreased inter-sister-chromatid distances were observed after DSB induction in chicken DT40 cells, consistent with the establishment of DI cohesion [43]. Moreover, × rays enhanced sister-chromatid alignment in plants [44]. Finally, ChIP followed by deep sequencing (ChIP-seq) revealed that IR triggered an Esco1-dependent increase in Smc3 acetylation and a genome-wide reinforcement of cohesin binding at pre-existing sites in human cells [45].

Future experiments are obviously needed to firmly establish DI cohesion as a conserved mechanism during DNA damage response in all eukaryotes. Moreover, even if DI cohesion exists in higher eukaryotes, the posttranslational modifications that regulate DI cohesion might be different from that of yeast. For example, DNA damage-induced Scc1 acetylation could not be detected in human cells [45]. The sites of Scc1 acetylation are also not conserved in human Scc1.

### 1.5. Cohesin and DNA damage checkpoint activation

In addition to its direct role in HR repair, cohesin is involved in DNA damage checkpoint activation (Figure 2). Several studies established the role of cohesin in intra-S-phase checkpoint activation in human cells [46-49]. In response to DNA damage [e.g. IR, UV, and hydroxyurea (HU)], ATM or ATR phosphorylated two residues, S957 and S966, of Smc1, and phosphorylation of these two sites was required for S-phase checkpoint activation. In addition to Smc1, Smc3 was also phosphorylated by ATM at S1083 in response to IR, and S1083 phosphorylation was similarly required for the intra-S phase checkpoint [50]. The Smc1/3 functions in the intra-S checkpoint are apparently mediated by the intact cohesin, not through a separate Smc1/3-containing recombination complex [51].

How cohesin phosphorylation activates intra-S phase checkpoint to block DNA synthesis in response to DNA damage remains unclear at present and awaits the identification of the downstream effectors of Smc1/3 phosphorylation. These phosphorylation events may directly recruit proteins essential for checkpoint activation to damage sites. Alternatively, phosphorylation of cohesin may affect its dynamic association with chromatin through regulating the ATPase activities of Smc1/3. This in turn may allow cohesin to act as barriers on chromatin and directly slow down DNA replication. Regardless of the mechanism, cohesin's role in S-phase checkpoint activation highlights a two-way crosstalk between cohesin and DNA replication.

Recently, cohesin has also been implicated in the G2/M DNA damage checkpoint in human cells [51]. Depletion of Scc1, but not sororin, by RNAi caused defective 53BP1 recruitment to DNA damage foci and weaker Chk2 activation in G2. Because sororin is required for functional cohesion, this result suggested that the G2/M checkpoint function of cohesin could be uncoupled from its function in sister-chromatid cohesion. Consistent with this notion, cohesin was also required for Chk2 activation in G1 prior to DNA replication (Figure 2).

### 1.6. Separase-mediated cohesin cleavage in DNA repair

An interesting study in *S. pombe *implicated a requirement for cohesin cleavage by separase in DNA repair [52]. It was shown that the separase inhibitor securin was essential for the proper repair of DNA damage induced by UV and IR. Expression of a non-cleavable Scc1 or inactivation of separase impaired DNA repair during G2, suggesting that the DNA repair functions of securin and separase acted through the cleavage of cohesin. Whether cohesin cleavage by separase is required for DNA repair in other organisms remains to be determined. It is also unclear how separase becomes active in G2 and how its activity is presumably restricted to DNA damage sites.

## 2. The Smc2/4 condensin complexes

Condensin complexes are five-subunit complexes that regulate chromosome organization and condensation during mitosis and meiosis in eukaryotic cells. They are responsible for folding chromatin fibers into highly compact chromosomes to ensure their faithful segregation. In vertebrates, there are two types of condensin complexes: condensin I and condensin II [6] (Table 1). Condensins I and II share two core subunits, Smc2/CAP-E and Smc4/CAP-C, but differ in the other three non-Smc subunits. Condensin I contains CAP-D2, CAP-H, and CAP-G while condensin II contains CAP-D3, CAP-H2, and CAP-G2 [53] (Figure 1). The two condensin complexes have apparently different roles in chromosome organization [54]. Depletion of condensin I produces a swollen chromosome shape while depletion of condensin II produces a curly shape. Depletion of both results in the formation of cloud-like chromosomes with a fuzzy appearance. In addition to condensins I and II, *C. elegans *has a specialized condensin-like Smc complex that regulates dosage compensation. This complex is composed of Smc2, an Smc4 variant called DPY-27, and three non-Smc proteins, DPY-26, DPY-28, and CAPG-1 [3,55].

### 2.1. Condensins in checkpoint activation and DNA repair

The first finding to reveal condensin's role in DNA repair came from a study in *S. pombe *[56]. In this study, Aono *et al. *isolated a temperature-sensitive mutant of *cnd2*, a non-Smc subunit in the condensin complex [56]. In addition to the expected mitotic chromosome condensation defects, this mutant exhibited elevated sensitivity to UV, HU, and methyl methanesulfonate (MMS), and a defect in Cds1 (the fission yeast ortholog of Chk2) activation. These results established a role of condensin in the replication checkpoint control and DNA repair. In a subsequent study, the same group identified a new condensin-binding protein called Cti1, using the hinge domain of Cut3 (Smc4) as the bait in a yeast two-hybrid screen [57]. Overexpression of Cti1 suppressed the UV and HU sensitivity of the Cnd2 mutant, suggesting that Cti1 positively regulated the DNA repair function of condensin.

The two condensin complexes in humans are also involved in DNA repair. Condensin I has been shown to play a role in DNA single-strand break (SSB) repair by interacting with the PARP1-XRCC1 complex [58,59]. Condensin I does not appear to play a significant role in DSB repair. By contrast, a recent study showed that condensin II was involved in HR repair of DSBs to maintain genome integrity [60]. Furthermore, depletion of condensin II only affected HR repair of IR-induced DSBs, but not the activation of the G2/M checkpoint.

### 2.2. Condensins and rDNA stability

Aside from its direct role in DNA repair to maintain genomic stability, condensin prevents unwanted intrachromosomal HR at the rDNA locus and controls rDNA stability in yeast [61-63]. Condensin regulates rDNA condensation during interphase upon nutrient starvation. This sub-chromosomal DNA compaction likely inhibits intrachromosomal HR at this locus, reduces the production of extrachromosomal rDNA circles, and protects the integrity of the rDNA array. In the absence of condensin, Rad52 improperly localizes to the nucleolus. Deletion of Rad52 rescues the cell lethality under nutrient starvation caused by condensin inactivation. Thus, condensin-dependent nucleolus exclusion of Rad52 provides one mechanism for the regulation of rDNA stability by condensin.

rDNA stability is critical for normal nucleolar function and ribosome biogenesis. Dysregulation of ribosome biogenesis has been linked to cancer and other human diseases. In the future, it will be important to determine whether the rDNA protection function of condensin is conserved in higher organisms, including humans. Along this vein, both *Xenopus *and human condensins have been shown to be associated with nucleolus during interphase [64,65].

## 3. The Smc5/6 complex

Unlike cohesin and condensins which have other major non-DNA repair functions in chromosome biology, the Smc5/6 complex is primarily required for DNA repair. The Smc5/6 complex is composed of Smc5, Smc6, and several non-SMC elements (Nse), including Nse1-6 (Table 1). Nse4 bridges the ATPase head domains of Smc5 and Smc6 (Figure 1), possibly in a manner similar to the role of Scc1 in linking Smc1 and Smc3. Nse1 interacts with Nse3, and both Nse1 and Nse3 bind to Nse4. Mms21/Nse2 does not bind to the ATPase head domains of Smc5/6, but interacts with the coiled-coil region of Smc5 [4,66]. Intriguingly, two of the Nse proteins have enzymatic activities. Nse1 contains a RING domain commonly found in ubiquitin ligases and forms an active ubiquin ligase with the MAGE (melanoma-associated antigen gene) protein Nse3 [67]. Mms21 contains an SP-RING domain and has small ubiquitin-like modifier (SUMO) ligase activity [68-70]. The SUMO ligase activity of Mms21 has been shown to be crucial for DNA damage repair and targets several substrates in different organisms from yeast to man.

### 3.1. The Smc5/6 complex and DSB repair by HR

The *Smc6 *gene was initially identified by its ability to correct the radiation sensitivity of the *rad18-X *mutant isolated in a screen for radiation-sensitive mutations in the fission yeast *S. pombe *[27,71,72]. Smc5 and Smc6 are essential genes in yeast. Hypomorphic alleles of the Smc5/6 complex exhibit defects in the repair of DNA damage caused by a broad spectrum of agents, including IR, UV, MMS, mitomycin C and HU [5,69,73-76]. In *S. pombe*, Smc6 is not only required for DSB repair induced by IR, but also is required for G2/M checkpoint activation [77]. Epistasis analysis further suggests a role of the Smc5/6 complex in HR, since *rad18, nse1, and nse2 *are epistatic with *rhp51 *(the fission yeast Rad51, a key HR protein) in response to IR [72,78].

**Figure 3:**
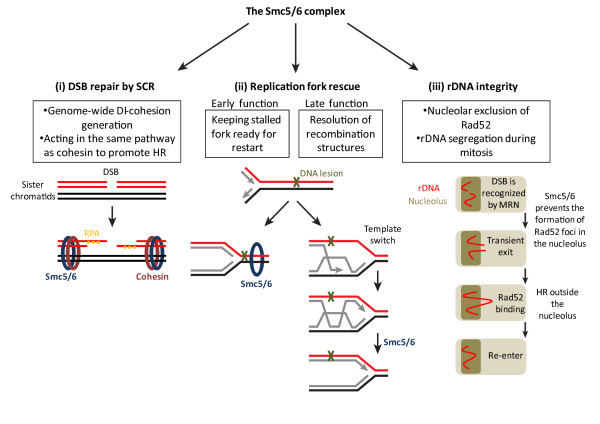
**Functions of the Smc5/6 complex in DNA repair and rDNA maintenance**. See text for details.

Consistent with these studies in the fission yeast, inactivation of the Smc5/6 complex in budding yeast, plants, chickens, and humans all results in sister-chromatid HR defects [34,44,79-81]. In keeping with the role of the Smc5/6 complex in HR, it is recruited to HO-induced DSBs in budding yeast and I-SceI-induced DSBs in human cells, as revealed by ChIP experiments [34,80,82]. Moreover, the Smc5/6 complex is only enriched at or around the HO-induced DSB in G2/M, but not in G1 when the sister chromatid is absent. Mre11, but not Mec1 and Rad53, are required for Smc6 recruitment to DSBs in yeast [82]. Similar to cohesin depletion, depletion of the Smc5/6 complex in human cells reduced SCE. Co-depletion of both cohesin and the Smc5/6 did not further reduce SCE, suggesting that they acted in the same pathway to promote sister-chromatid HR [34] (Figure 3). Future studies are required to address the mechanisms by which the Smc5/6 complex promotes HR between sister chromatids.

### 3.2. The Smc5/6 complex and stalled replication forks

A second function of the Smc5/6 complex in DNA repair is the repair of collapsed replication forks [83]. Smc6 localizes to collapsed replication forks in budding yeast [82]. Inactivation of Smc5/6 caused accumulation of X-shaped HR intermediates that could be formed by the regression of stalled replication forks in rDNA. Furthermore, the SUMO ligase activity of Mms21 is required for preventing the accumulation of the X-shaped DNA molecules at damaged replication forks [84], although the relevant substrate of Mms21 in this process is unknown. Overexpression of BRC1 (a BRCT domain protein required for DNA repair during S phase) or the bacterial resolvase RuvA rescued the replication-arresting defects of *nse5, nse6*, and *smc6 *mutants in *S. pombe *[76,85,86]. The structure-specific endonucleases Slx1/4 and Mus81/Eme1 are required for the BRC1-mediated suppression of Smc5/6 mutant phenotypes. Moreover, inactivation of the Mph1 helicase suppressed the accumulation of aberrant recombination intermediates in *smc6 *and *mms21*/*nse2 *mutants in *S. cerevisiae *[87]. Smc5/6 has also been shown to facilitate the resolution of sister-chromatid linkages during mitosis [88,89]. Finally, the Smc5/6 complex is required for loading RPA and Rad52 onto stalled replication forks to maintain them in recombination-competent configurations [90]. Collectively, these investigations indicate that the Smc5/6 complex promotes HR-dependent rescue of stalled replication forks by stabilizing them in recombination-component configurations and by facilitating the resolution or preventing the formation of certain recombination intermediates (Figure 3).

### 3.3. The Smc5/6 complex and rDNA integrity

Similar to condensin, the Smc5/6 complex is required for the maintenance of rDNA stability in budding yeast (Figure 3). The Smc5/6 complex accumulates at rDNA regions in budding yeast [82]. It is enriched in the nucleolus [91]. Inactivation of Smc5 or the Mms21 SUMO ligase activity results in fragmented and irregularly shaped nucleoli [70,92], indicating a role of the Smc5/6 complex in maintaining rDNA integrity. The repair of DSBs in the rDNA locus occurs outside the nucleolus. To preserve the repetitive sequence of the rDNA array, DSB recognition, end resection, and RPA binding happen within the nucleolus. On the other hand, binding of the key downstream HR proteins Rad51 and Rad52 to these DSBs are excluded from the nucleolus. Cells harboring mutations in the Smc5/6 complex exhibit Rad52 foci at the DSBs in the nucleolus and elevated numbers of extrachromosomal rDNA circles [93]. These findings suggest that the Smc5/6 complex mediates the nucleolar exclusion of Rad52, thereby suppressing the recombinational loss of rDNA repeats to ensure rDNA stability (Figure 3).

## Conclusions

The Smc family of proteins has critical roles in the DNA damage response of organisms from yeast to man. The Smc1/3 cohesin complex promotes DNA double-strand break (DSB) repair through homologous recombination (HR) between sister chromatids, presumably by holding sister chromatids in proximity to help strand invasion. Cohesin is also required for DNA damage checkpoint activation. The condensin complexes are required for DNA damage checkpoint activation, DNA repair, and rDNA stability. The Smc5/6 complex facilitates DSB repair through HR between sister chromatids and does so in the same pathway as cohesin. The Smc5/6 complex has additional roles in DNA repair, including resolution of collapsed replication forks and rDNA maintenance.

Many outstanding questions still remain in this area. First, the detailed molecular mechanisms by which the Smc proteins mediate DNA repair are not understood. In particular, in the cases of cohesin and condensin, it is unclear whether their DNA repair functions are separable from their major functions in sister-chromatid cohesion and chromosome condensation. Second, more needs to be learned about how the DNA repair functions of the Smc complexes are regulated during the cell cycle. Finally, the coordination and crosstalk among the three Smc complexes in the DNA damage response need to be further examined. Both cohesin and the Smc5/6 complex act in the same pathway to repair DSBs through HR between sister chromatids. How do they communicate with each other? Likewise, both condensin and the Smc5/6 complex are required for rDNA stability in yeast. Is this function of condensin and Smc5/6 conserved in higher eukaryotes? Do these two complexes function in the same or different pathways? Future studies aimed at addressing these questions will greatly advance our understanding of the molecular mechanisms underlying chromosome maintenance and genome stability.

Mutations of the Smc complexes and their regulators have been linked to human diseases, including cancer. A better understanding of how these complexes protect genomic stability will help us understand the molecular basis of disease phenotypes and may ultimately lead to strategies that exploit the dysregulation of the Smc proteins to treat these human diseases.

## Competing interests

The authors declare that they have no competing interests.

## Authors' contributions

NW prepared the initial draft of the paper. HY modified and finalized the paper. All authors read and approved the final manuscript.
